# HtrA2 suppresses autoimmune arthritis and regulates activation of STAT3

**DOI:** 10.1038/srep39393

**Published:** 2016-12-23

**Authors:** Seung Hoon Lee, Young-Mee Moon, Hyeon-Beom Seo, Se-Young Kim, Eun-Kyung Kim, Junyeong Yi, Min-Kyung Nam, Jun-Ki Min, Sung-Hwan Park, Hyangshuk Rhim, Mi-La Cho

**Affiliations:** 1 The Rheumatism Research Center, Catholic Research Institute of Medical Science, The Catholic University of Korea, Seoul, South Korea; 2 Laboratory of Immune Network, Conversant Research Consortium in Immunologic disease, College of Medicine, The Catholic University of Korea, Seoul, South Korea.; 3 Department of Biomedicine and Health Sciences, College of Medicine, The Catholic University of Korea, Seoul 137-701, Republic of Korea; 4 Department of Medical Life Sciences, College of Medicine, The Catholic University of Korea, Seoul 137-701, Republic of Korea

## Abstract

Rheumatoid arthritis (RA) is an autoimmune disease that is related to the induction of T helper (Th)17 cells, which secrete interleukin-17, and activation of the signal transducer and activator of transcription (STAT) 3. The expression of high-temperature requirement protein A (HtrA) 2, a serine protease involved in apoptosis, was decreased in RA patients nonresponsive to drug treatment of RA. The aim of this study was to determine whether overexpression of HtrA2 has a therapeutic effect on RA. Th17 differentiation, osteoclastogenesis, and lymphocyte activation are increased in motor neuron degeneration (mnd)2 mice, which lack HtrA2 activity because of a missense mutation (Ser276Cys) in the protease domain of HtrA2. The inhibitor of HtrA2 also increased Th17 differentiation. On the other hand, HtrA2 induced cleavage of STAT3 and overexpression of HtrA2 attenuated CIA in a mouse model. HtrA2 overexpression inhibited plaque development as well as the differentiation of Th17 in ApoE^−/−^ mice after immunization with proteoglycans to induce a hyperlipidemia-based RA animal model. The therapeutic function of HtrA2 in inflammatory diseases is linked with Th17 development and the STAT3 pathway in splenocytes. These results suggest that HtrA2 participates in immunomodulatory activity where the upregulation of HtrA2 may shed light on therapeutic approaches to RA and hyperlipidemia.

Rheumatoid arthritis (RA) is a systemic autoimmune disorder involving chronic inflammation. RA is characterized by an excessive inflammatory response and deforming polyarthritis, activation of T cells. Interleukin (IL)-17 produced by T helper (Th) 17 cells increases in the peripheral blood of patients with RA^1^. Additionally, Th17 cells exacerbate tissue destruction by inducing chronic inflammation in patients with RA^2^. The expression of proinflammatory cytokines is related to the pathogenesis of RA where several proinflammatory cytokines participate in the augmentation of RA^3^. In particular, IL-17 leads to a chronic immune inflammatory response in patients with RA^2^, and its production is upregulated in patients with RA^4^.

Signal transducer and activator of transcription (STAT) 3 is a DNA binding transcription factor that controls the production of a number of cytokines and T cell lineage. Activation of STAT3 induces the production of IL-17^5^,^6^ and inflammatory CD4^+^ T cells, such as Th17^6^,^7^. Indeed, the differentiation of Th17 is regulated directly by STAT3^8^. This in turn allows for STAT3 being a potential target for RA therapy where inhibiting it reduced Th17 differentiation in an experimental autoimmune arthritis model^9^,^10^.

A considerable proportion of patients with RA fail to respond to drug treatment. For instance, 30–40% of patients with RA receiving anti-tumor necrosis factor (TNF)-α therapy did not respond^11^. Several TNF-α blocker including infliximab do not have therapeutic effects in one-third of patients with RA^12^. In addition, these patients have an increased possibility of not responding to other biologics^13^.

High-temperature requirement protein A (HtrA) 2 is a serine protease that is released into the cytosol during apoptosis and is involved in neurodegeneration^14^. There is evidence that HtrA2 deficiency or a point mutation in the gene can result in neurodegeneration^14^,^15^,^16^. In RA patients, HtrA2 may be related to nonresponsiveness to RA drug treatment. It is well documented that gene expression of HtrA2 in peripheral blood mononuclear cells of nonresponders to methotrexate (MTX) is significantly decreased compared with that in responders; therefore, HtrA2 is an attractive candidate to test in nonresponders to MTX^17^.

Motor neuron degeneration (mnd)2 mice lack HtrA2 activity because of the missense mutation Ser276Cys in the protease domain of HtrA2^18^. It has been documented that loss of HtrA2 function results in dysfunction including cell death^19^. Indeed, mnd2 mice show tendrils in the spleen and thymus, and death occurs 40 days after birth^20^. It is also widely believed that this specific gene mutation causes disproportionate inflammation and untimely death^21^.

We hypothesized that HtrA2 is associated with RA pathogenesis mediated by STAT3 and Th17. The current study was performed to identify whether HtrA2 could be a therapeutic target in RA. First, we investigated the role of HtrA2 on STAT3 expression and Th17 differentiation. Second, we analyzed the therapeutic effect of HtrA2 expression in a model of experimental autoimmune arthritis. Finally, we examined whether HtrA2 inhibits the development of plaque and Th17 differentiation in APOE knockout mice immunized with a proteoglycan to induce a hyperlipidemia-based animal model of RA.

## Results

### Excessive activation of T cells in mnd2 mutation

**Figure 1:**
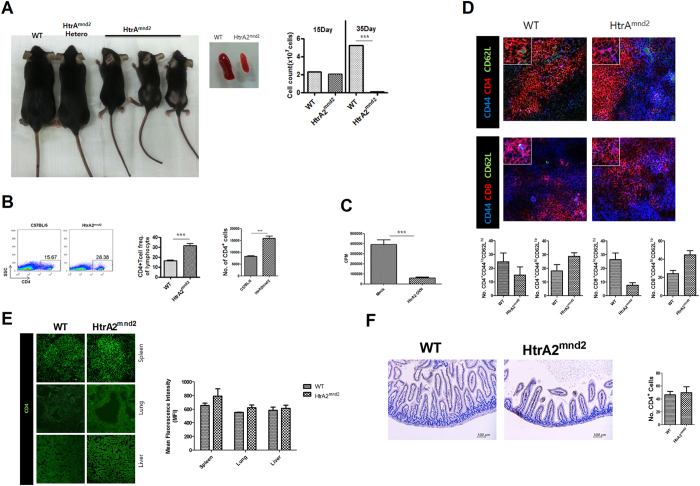
HtrA2 regulates T cell activation. (**A**) WT and HtrA2^mnd2^ phenotype mice. (**B**) The WT and HtrA2^mnd2^ mutant mice CD4^+^ T cell populations were analyzed by flow cytometry (n = 6). (**C**) A T cell proliferation response assay (MLR) was conducted to measure T cell activity of LBRM transfected with HtrA2 or mock vector (n = 3). (**D**) CD4^+^CD44^+^CD62L^+^ and CD4^+^CD44^+^CD62L^+^ T cell populations in WT and HtrA2^mnd2^ mutant mice were analyzed by confocal scanning microscopy (n = 3). (**E**) CD4^+^ T cell populations in spleen, lung, and liver from WT and HtrA2^mnd2^ mutant mice were examined by confocal scanning microscopy (n = 3). (**F**) The CD4^+^ T cell populations in colon from WT and HtrA2^mnd2^ mutant mice were measured by immunocytochemistry (n = 3). Data are mean ± standard deviation of three independent experiments (***P < 0.01).

HtrA2^mnd2^ is single point mutation of HtrA2 causing an untimely death^20^ and dysregulated inflammation^21^. We hypothesized that HtrA2^mnd2^ may be involved in an excessive immune response. HtrA2^mnd2^ caused an abnormal small body size and a smaller spleen with fewer cells (Fig. 1A). On the other hand, CD4^+^ cells in the spleen of HtrA2^mnd2^ increased significantly compared to that of wild type (WT) (Fig. 1B). We also investigated whether HtrA2 could downregulate the activation of T cells. In the mixed lymphocyte reaction (MLR), HtrA2 overexpression in LBRM cells significantly decreased T cell proliferation compared with that induced by mock vector in LBRM cells (Fig. 1C). We also found that the numbers of naïve T cells (both CD4^+^ and CD8^+^) were reduced but those of memory T cells were increased in spleens from HtrA2mnd2 mice (Fig. 1D). CD4^+^ T cells were also slightly higher in spleen, lung, liver, and gut from HtrA2^mnd2^ mice than in the same tissues from WT mice (Fig. 1E and F).

### Regulation of STAT3 production by HtrA2 expression

**Figure 2:**
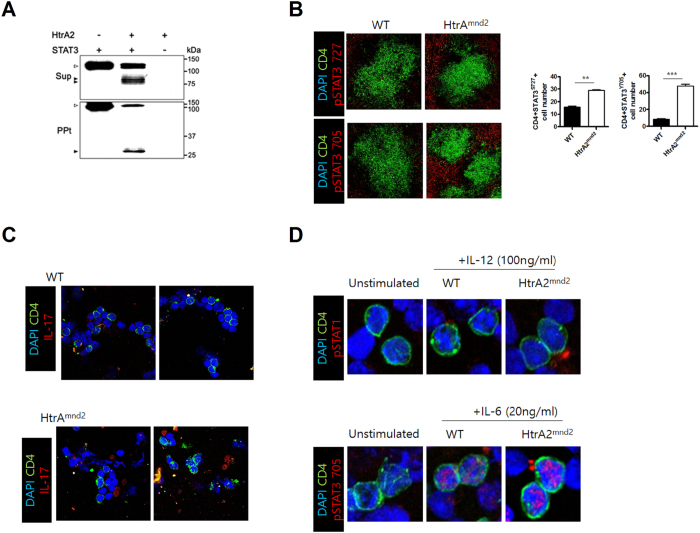
STAT3 is directly cleaved by HtrA2, and HtrA2 deficiency induces excess STAT3 and IL-17 expression. (**A**) STAT3 and HtrA2 were expressed as GST-fusion proteins and purified by selective binding to glutathione-Sepharose 4B beads. The proteins were incubated for 16 hr at 37 °C. The reaction mixtures were separated into a supernatant (Sup) and GST proteins bound to beads (precipitate, PPt). The fractions were resolve by 15% SDS-PAGE and analyzed by IB assay with the STAT3 antibody. Open and closed arrowheads indicate STAT3 (118 kDa) and cleaved STAT3 (81, 75, and 28 kDa), respectively. (**B**) pSTAT3 Tyr705 and pSTAT3 Ser727 expression in splenocytes from WT and HtrA2^mnd2^ mutant mice (original magnification, ×40, n = 6) The number of cells was counted in four independent quadrants. (**C**) CD4 and IL-17 expression in splenocytes of WT and HtrA2^mnd2^ mutant mice. (D) CD4^+^pSTAT1^+^ and CD4^+^pSTAT3 705^+^ expression in splenocytes from WT and HtrA2mnd2 mutant mice. Data are mean ± standard deviation of three independent experiments (***P < 0.01).

To identify whether HtrA2 reduces STAT3 expression associated with inflammation^22^,^23^, we performed an *in vitro* cleavage assay using GST-fusion proteins of STAT3 and HtrA2. We found that STAT3 was degraded by HtrA2 (Fig. 2A). However, the number of pSTAT3 705^+^ and pSTAT3 727^+^ cells significantly increased in the spleen of HtrA2^mnd2^ mice compared to WT (Fig. 2B). CD4^+^IL-17^+^ cells were also promoted in splenocytes of HtrA2^mnd2^ mice compared to that in WT (Fig. 2C). In addition, the level of pSTAT3 was increased by IL-6 stimulation, but IL-12 failed to increase pSTAT1 expression (Fig. 2D).

### Excess IL-17 production induced by HtrA2 deficiency

**Figure 3:**
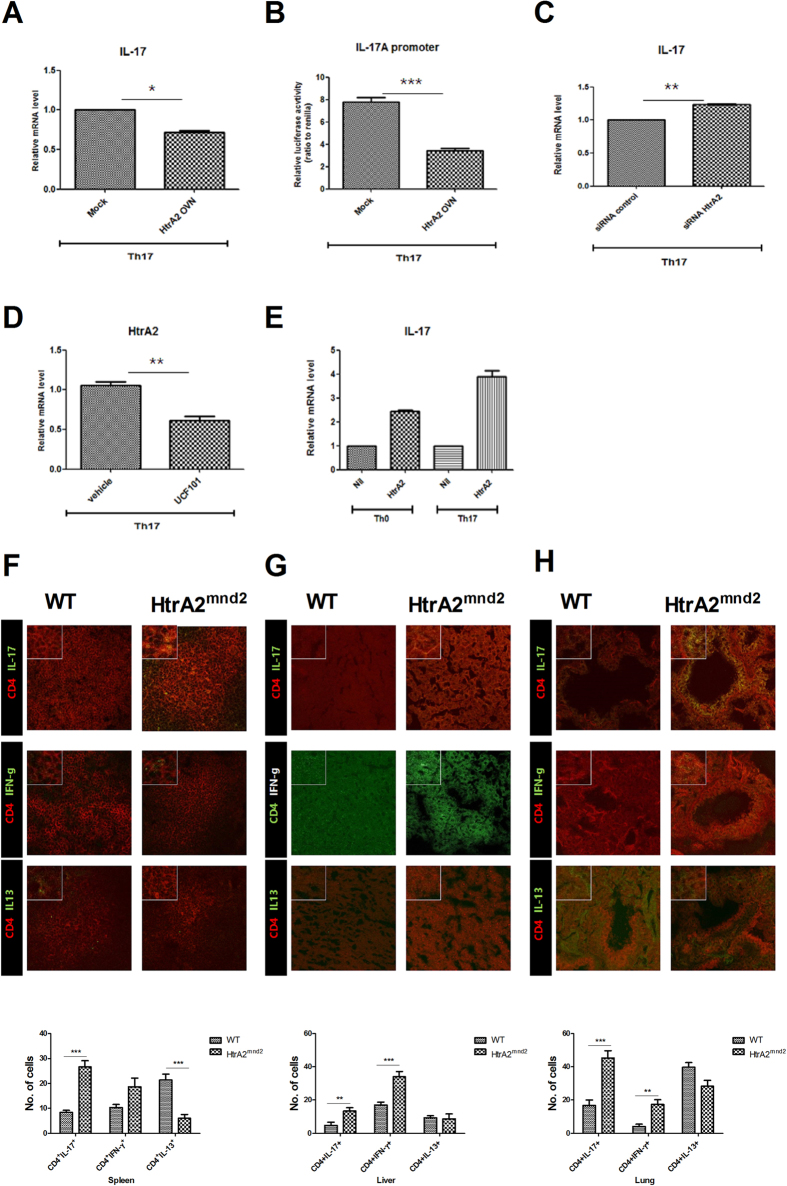
IL-17 gene expression is regulated by HtrA2. (**A**) Relative mRNA expression of IL-17 in mice LBRM transfected by HtrA2 or mock vector and incubated for 72 hours under Th17 cell-polarizing conditions (stimulation only with anti-CD3 and anti-CD28 with TGF-β and IL-6) was measured by real-time PCR. (**B**) LBRM transfected with the HtrA2 or mock vector and the Il17a promoter was cultured for 72 hours under Th17 cell–polarizing conditions. Luciferase activity was examined using a dual-luciferase reporter assay system. (**C**) Relative IL- mRNA 17 expression was analyzed by real-time PCR using HrA2 siRNA-transfected LBRM cultured for 72 hours under Th17 cell-polarizing conditions. (**D**) Relative HtrA2 mRNA expression was analyzed by real-time PCR using UCF101 treated LBRM cultured for 72 hours under Th17 cell-polarizing conditions. (**E**) Relative IL-17 mRNA expression was analyzed by real-time PCR using UCF101 treated LBRM cultured for 72 hours under Th0 cell conditions (stimulation only with anti-CD3 and anti-CD28 without added cytokines) or Th17 cell-polarizing conditions. (**F–H**) CD4^+^IL-17^+^, CD4^+^IFN-γ^+^, and CD4^+^IL-13^+^ T cell populations in spleen, liver, and lung from WT and HtrA2^mnd2^ mutant mice were measured by confocal scanning microscopy (n = 3). The number of cells was counted in four independent quadrants. Data are mean ± standard deviation of three independent experiments (*P < 0.05, **P < 0.03, ***P < 0.01, n = 3).

As STAT3 directly regulates IL-17 expression^6^, we analyzed IL-17 levels mediated by HtrA2. Gene expression of IL-17 in LBRM decreased significantly by overexpressing HtrA2 (Fig. 3A). IL-17A promoter activity also decreased significantly by HtrA2 overexpression (Fig. 3B). In contrast, siRNA HtrA2 increased IL-17 mRNA significantly (Fig. 3C). We also found that the HtrA2 inhibitor, UCF101, significantly decreased the level of HtrA2 mRNA but enhanced that of IL-17 (Fig. 3D and E). In addition, CD4^+^IL-17^+^ and CD4^+^IFN-γ^+^ cells were increased in spleen, liver, and lung from HtrA2^mnd2^ compared with WT. In contrast, CD4^+^IL-13^+^ cells were reduced in these tissues from HtrA2^mnd2^ mice compared with WT (Fig. 3F–H).

### HtrA2 deficiency and mnd2 mutation results in Th17 differentiation and osteoclastogenesis

**Figure 4:**
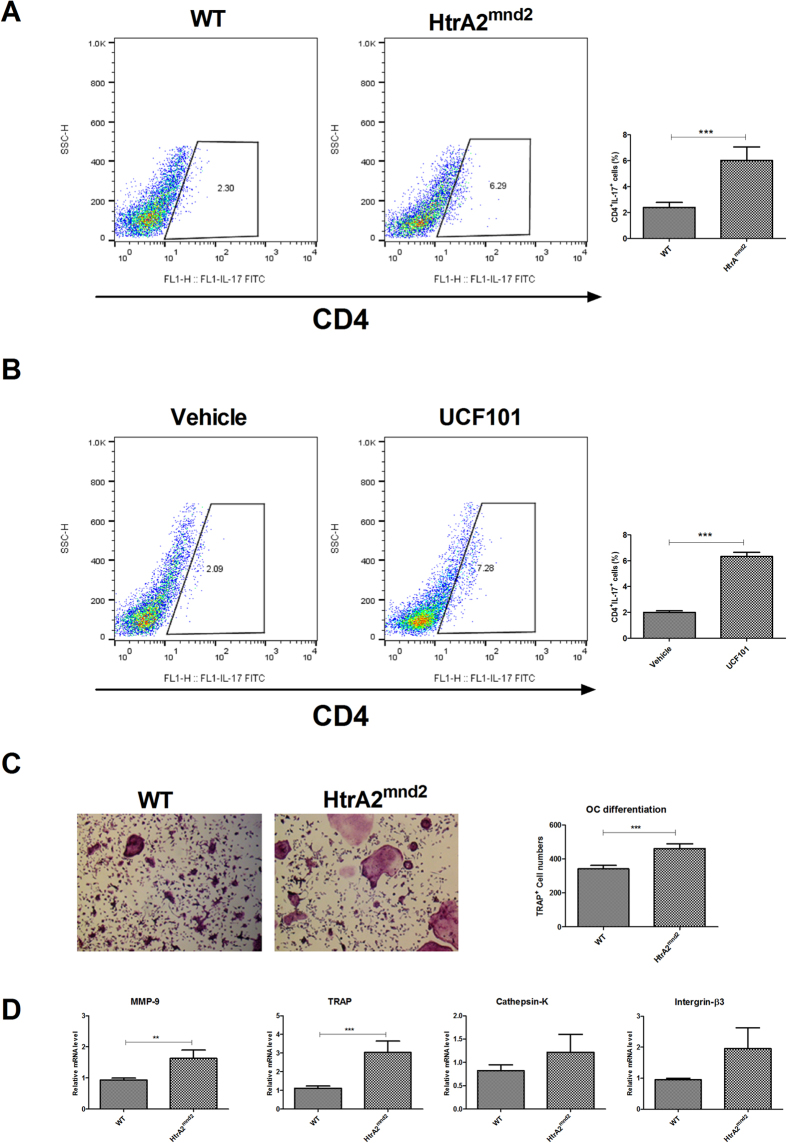
Loss of HtrA2 induces Th17 differentiation and osteoclast formation. (**A and B**) IL-17A expression in CD4^+^ T cells from WT and HtrA2^mnd2^ mutant mice or CD4^+^ T cells treated with UCF101 or vehicle and incubated for 3 days under Th17 cell-polarizing conditions (stimulation only with anti-CD3 and anti-CD28 with TGF-β and IL-6) was measured by flow cytometry (n = 6). (**C**) Bone marrow cells from WT and HtrA2^mnd2^ mutant mice were cultured with M-CSF (10 ng/ml) and RANKL (50 ng/ml). Cells were fixed and stained for TRAP, and the number of TRAP^+^ cells was counted using a light microscope (original magnification, ×100 n = 6). (**D**) Relative mRNA expression of osteoclastogenic markers was measured by real-time PCR (n = 6). Data are mean ± standard deviation of three independent experiments (**P < 0.03, ***P < 0.01).

STAT3 directly controls development of Th17, which induces osteoclastogenesis^6^,^24^. We analyzed Th17 differentiation and osteoclastogenesis to determine whether HtrA2 can modulate Th17 differentiation and osteoclastogenesis. CD4^+^IL17^+^ cells of HtrA2^mnd2^ significantly increased under the Th17 condition compared to those in WT (Fig. 4A). UCF101 also significantly increased CD4^+^IL17^+^ cells compared to that in the vehicle (Fig. 4B). HtrA2^mnd2^ significantly increased osteoclastogenesis compared to that of WT (Fig. 4C). mRNA levels of tartrate-resistant acid phosphatase (TRAP), matrix metalloproteinase9, cathepsin-K, and integrin-β3 involved in osteoclastogenesis were promoted in HtrA2^mnd2^ compared to those in WT (Fig. 4D).

### HtrA2 overexpression attenuates CIA severity reducing Th17 differentiation

**Figure 5:**
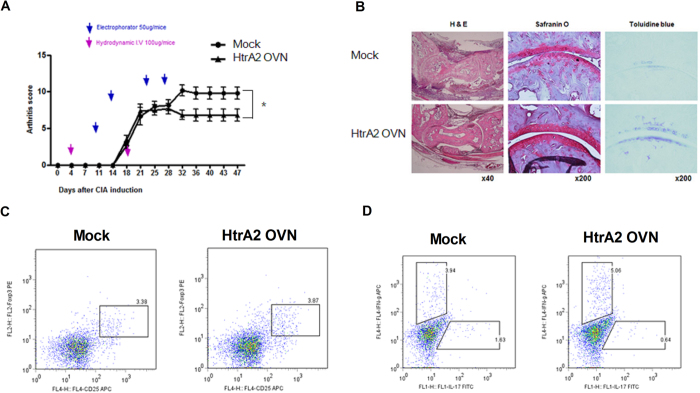
HtrA2 attenuates CIA progression. HtrA2 or mock vector was administered systemically one time weekly into CIA induced mice. Mice were sacrificed on day 47 after the first immunization. (**A**) The arthritis scores of CIA mice treated with HtrA2 or mock vector. (**B**) Joint tissues from HtrA2 or mock vector-treated CIA mice were stained with hematoxylin-eosin, Safranin-O, and toluidine blue (n = 6). (**C and D**) Differentiation of Treg, Th1, and Th17 cells in spleens of CIA mice treated with HtrA2 or mock vector.

To determine whether HtrA2 has therapeutic activity in RA, we measured the development of CIA in mice injected with either the HtrA2 overexpressing or mock vector once weekly from day 7 after the first immunization. HtrA2 overexpression significantly reduced CIA severity compared to that of mock (Fig. 5A). A histological analysis indicated that HtrA2 overexpression attenuated inflammation and bone and cartilage damage (Fig. 5B). The overexpression of HtrA2 decreased Th17 differentiation in the spleens of mice with CIA (Fig. 5C). Regulatory T (Treg) cell differentiation was not changed in CIA-induced mice injected with the HtrA2 overexpressing vector (Fig. 5D).

### HtrA2 overexpression decreased atherosclerotic plaque burden and inflammation in hyperlipidemia-based mouse RA

**Figure 6:**
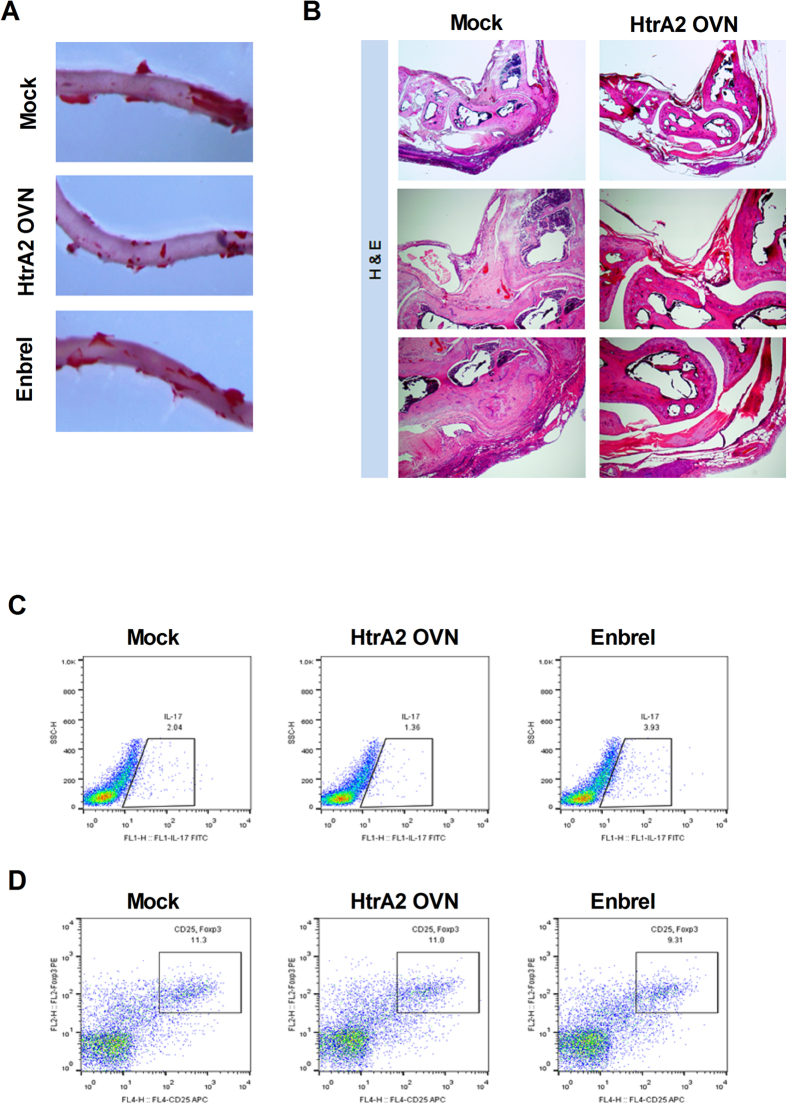
HtrA2 decreases atherosclerotic plaque burden and joint inflammation in mice with hyperlipidemia based RA. (**A**) Aorta stained with Oil Red-O to label the plaques (original magnification, ×100, n = 3). (**B**) Joints stained with H&E to detect infiltration of immune cells (original magnification, ×40 or ×100, n = 3). (**C and D**) Differentiation of Th17 and Treg in spleens of mice with hyperlipidemia based RA treated with Enbrel, HtrA2 or Mock vector.

Metabolic disorders are correlated with non-response to RA treatment^25^. As ApoE^−/−^ mice generate atherosclerotic lesions repeatedly at susceptible locations, particular at branch points of the aorta^26^,^27^, we examined whether overexpressing HtrA2 is therapeutic and reduces aortic plaque progression in ApoE^−/−^ mice immunized with proteoglycan to induce a hyperlipidemia-based RA animal model. Results showed that HtrA2 overexpression inhibited aortic plaque formation compared to that of mock and enbrel (Fig. 6A). HtrA2 overexpression also decreased the infiltration of immune cells and bone destruction (Fig. 6B). There was a reduction in Th17 differentiation, however, Treg differentiation remained unchanged with HtrA2 overexpression (Fig. 6D).

## Discussion

HtrA2 has been investigated primarily as a serine protease with neurodegeneration and apoptosis functions 14,16. HtrA2 has been suggested to be a candidate associated with non-response to drug treatments for RA^17^. However, minimal information is available regarding the therapeutic effect of HtrA2 in RA. Here, we investigated the therapeutic function of HtrA2 and identified its remedial process in RA.

The most remarkable finding of this study was that HtrA2 ameliorated RA by inhibiting STAT3. As STAT3 enhances IL-17 production and Th17 differentiation^5^,^6^,^7^, inhibiting STAT3 is a promising strategy for RA therapy. In this study, we found that HtrA2^mnd2^ significantly increased pSTAT3 expression, whereas HtrA2 induced STAT3 degradation *in vitro*. As well, HtrA2 revealed curative activity in a CIA and hyperlipidemia-based RA. To our knowledge, this is the first study suggesting the use of HtrA2 in RA therapy by inhibiting STAT3. Furthermore, results found that immoderate T cell activation and proliferation of CD4^+^ T cells, which can induce uncontrolled inflammation such as an inflammatory storm, and may be involved in early death^20^.

Th17 release of IL-17 has been shown to be positively correlated with the progression of RA. As IL-17 upregulates the inflammatory response^28^,^29^,^30^, Th17 and IL-17 exacerbates inflammation in RA^2^,^31^,^32^. Th17 results in osteoclastogenesis and bone annihilation as RA progresses^24^,^33^. The release of IL-17 from Th17 cells is controlled by IL-13, which can inhibit the expression of proinflammatory cytokines^34^,^35^. Our data indicates that HtrA2 overexpression decreased the activation of T cells and IL-17 gene expression and promoter activity, however, the HtrA2 inhibitor and HtrA2^mnd2^ increased IL-17 mRNA levels as well as the proliferation of Th17 cells. Supporting this, CD4^+^IL-17^+^ and CD4^+^IFN-γ^+^ cells were also increased but CD4^+^IL-13^+^ cells were reduced in several tissues from HtrA2^mnd2^mice. Additionally, osteoclastogenesis of HtrA2^mnd2^ increased significantly compared to that of WT. As Th17 and IL-17 cause excessive inflammation during RA development, our results suggest a novel therapeutic function of HtrA2 as a treatment for RA.

CD4^+^ and CD8^+^ T cells are involved in maintaining homeostasis of the immune system^36^. Because naïve T cells (CD62L^hi^CD44^lo^) differentiate into memory T cells (CD62L^lo^CD44^hi^) after exposure to antigen, memory T cells have previously encountered and responded to antigen^37^. Our observation that naïve T cells were decreased whereas memory T cells were increased in HtrA2^mnd2^ mice suggests that HtrA2 may play an important role in the immune response.

Metabolic disorders are correlated with being non-responsive to RA treatment^25^ and may indicate similar effects to other RA drugs^13^. HtrA2 has been pointed out as a significant target for non-response to MTX^17^. Moreover, HtrA2 mRNA levels in peripheral blood mononuclear cells and CD4^+^ T cells of healthy individuals and patients with RA from the National Center for Biotechnology Information Gene Expression Omnibus database (GSE15573 and GSE4588) were analyzed. Here, HtrA2 gene expression increased in patients with RA compared to that in healthy controls (Supplementary Figure 1A). However, HtrA2 mRNA expression in patients with familial hypercholesterolemia, a genetic disorder of uncontrolled high cholesterol level related to primary hyperlipidemia^38^, decreased compared to that of controls (Supplementary Figure 1B). Additionally, HtrA2 gene expression levels in patients with RA within the GSE58795 database inclusive to clinical information was analyzed and found that HtrA2 expression of non-responders and moderate responders was significantly downregulated compared to that of responders (Supplementary Figure 1B). These results suggest that HtrA2 expression may be increased to protect against RA pathogenesis in a way similar to that of IL-10^39^. HtrA2 may have a remedial function of reducing plaque formation in the aorta and differentiation of Th17 in hyperlipidemia-based RA. Thus, an HtrA2 deficiency may have no response to RA associated with a metabolic disorder.

Granulocyte-macrophage colony-stimulating factor (GM-CSF) is an immune modulatory cytokine that plays a key role in immune tolerance. There is evidence that GM-CSF ameliorates autoimmunity^40^,^41^. GM-CSF also increases differentiation of Treg cells, thereby reducing the severity of autoimmune disease^42^. However, GM-CSF can also mediate excessive inflammation and exacerbate autoimmune diseases including RA^34^. Particularly relevant to this study is the demonstration that GM-CSF deficiency protected against CIA progression^41^.

Eye-mediated immune tolerance may also be a good strategy for CIA treatment. It has been suggested that eye-induced immune tolerance to type II collagen has therapeutic benefit in an arthritis model^44^. CD8^+^ Tregs also perform a therapeutic function in CII-induced disease by inducing eye-induced tolerance^45^. Therefore, the therapeutic activity of HtrA2 in CIA may be related to induction of immune tolerance. However, further study is required to identify whether GM-CSF expression and eye-mediated immune tolerance are affected in CIA mice treated with HtrA2.

Diminished HtrA2 expression may be involved in the non-responsiveness to drugs for treating RA, however, there are few data available regarding the anti-arthritic effects of HtrA2. The new identified HtrA2 functions demonstrate its large role in ameliorating RA progression, which may shed new light on treating non-responders to medication. We have shown that HtrA2 suppressed Th17 differentiation and IL-17 expression by inhibiting STAT3. These preliminary data suggests that HtrA2 could be a crucial candidate for treating patients with RA including non-responders.

## Materials and Methods

### Animals

Male DBA1/J (Orient, Seoul, Korea) and apolipoprotein E deficient (ApoE^−/−^) mice (Jackson Laboratories, Bar Harbor, ME, USA) at 6–8-weeks-old were maintained in groups of five in polycarbonate cages in a specific pathogen-free environment. They were fed standard mouse chow (Ralston Purina, Gray Summit, MO, USA) and water *ad libitum*. Mice harboring the mutant motor neuron degeneration (mnd) 2 on a C57BL/6 mice background were obtained from Professor Hyangshuk Rhim (Department of Medical Life Sciences, College of Medicine, The Catholic University of Korea, Seoul, Republic of Korea). Heterozygous mnd2 (HT, mnd2/+) males were crossed with HT females 3–6 months of age to generate littermates of all three genotypes: wild-type (WT, +/+), HT, and homozygous mnd2 (mnd2/mnd2). The genotypes of the mice were analyzed by the polymerase chain reaction-restriction fragment length polymorphism (PCR-RFLP) method described previously^46^.

### Ethics statement

The Animal Care Committee of The Catholic University of Korea approved the experimental protocol. All experimental procedures were evaluated and carried out in accordance with the protocols approved by the Animal Research Ethics Committee at the Catholic University of Korea. All procedures performed followed the ethical guidelines for animal studies.

### Type II collagen immunization to induce arthritis

Collagen induced arthritis (CIA) was induced in DBA1/J mice (n = 10/group) by injecting 100 μg bovine type II collagen (Chondrex Inc., Redmond, WA, USA) dissolved overnight in 0.1 N acetic acid (4 mg/ml) in complete or incomplete Freund’s adjuvant (Chondrex) via the base of the tail. The immunization was performed intradermally.

### Clinical scoring of arthritis

Peripheral joints were examined for arthritis visually twice weekly. Arthritis was graded based on the method of Williams *et al*.^47^ with the following five grades: 1) Grade 0: no evidence of erythema or swelling; 2) grade 1: erythema and mild swelling confined to the mid-foot (tarsals) or ankle joint; 3) grade 2: erythema and mild swelling extending from the ankle to the mid-foot; 4) grade 3: erythema and moderate swelling extending from the ankle to the metatarsal joints; and 5) grade 4: erythema and severe swelling encompassing the ankle, foot, and digits. The final value represented the average index from all four legs recorded by two independent observers.

### Induction of hyperlipidemia-based RA and preparation of atherosclerotic lesions

ApoE^−/−^ mice were maintained in a temperature controlled (23 ± 2 °C) room. ApoE^−/−^ strain mice were injected with proteoglycan. The ApoE^−/−^ mice were fed a western type diet (Teklad Adjusted Calories Western-type diet), containing 21% fat by weight (0.15% by weight cholesterol and 19.5% by weight casein without sodium cholate) for 16 weeks. The aorta was uncovered longitudinally from the aortic root to the iliac bifurcation. The stained aorta tissues were kept in fixation solution until images were captured.

### Injection of agents

Eight days after immunization, mice with autoimmune arthritis were injected intravenously with 100 μg HtrA2 or mock vector in 2 ml saline over a 10-sec period. The same mice received an intramuscular injection of 50 μg HtrA2 or mock vector in the leg using electrical stimulation (electroporation). Enbrel (Pfizer, Berlin, Germany) was injected into ApoE^−/−^ mice. The injection was conducted subcutaneously.

### HtrA2 siRNA transfection

Small interfering RNA (siRNA) constructs for HtrA2 siRNA and non-targeting siRNA (Dharmacon, Lafayette, CO, USA) were obtained using siGENOME SMARTpool reagents (Dharmacon). siRNA was transfected using the Amaxa 4D-nucleofector X unit and the DN-100 program according to the manufacturer’s recommendations (Lonza, Cologne, Germany).

### Intracellular staining for flow cytometry

Before cell staining, differentiated CD4^+^ T cells were stimulated with 25 ng/ml phorbol myristate acetate and 250 ng/ml ionomycin (both from Sigma, St. Louis, MO, USA) in the presence of GolgiStop (BD Pharmingen, San Diego, CA, USA) for 4 hours. The cells were stained with anti-mouse CD4 peridin chlorophyll protein (PerCP), anti-mouse CD25 allophycocyanin (APC), anti-mouse IL-17 fluorescein isothiocyanate (FITC), and anti-mouse FOXP3 phycoerythrin (PE) (eBiosciences, San Diego, CA, USA) followed by fixation and permeabilization with a Foxp3 staining buffer kit according to the manufacturer’s instructions to examine intracellular cytokines. All samples operated on a FACSCalibur (BD Pharmingen) and data were analyzed using FlowJo software (Tree Star, Ashland, OR, USA).

### Confocal microscopy

Tissue cryosections (7 μm thick) were fixed with acetone and stained with FITC-, PE-, PerCP-Cy5.5-, or APC-conjugated monoclonal antibodies against mouse CD4, pSTAT3 (Tyr 705, Ser 727), IL-17, and FOXP3 (eBioscience). After an overnight incubation at 4 °C, the stained sections were visualized by confocal microscopy (LSM 510 Meta; Zeiss, Göttingen, Germany).

### Transfection

The HtrA2 vector was obtained from Professor Hyangshuk Rhim (Department of Medical Life Sciences, College of Medicine, The Catholic University of Korea, Seoul, Republic of Korea) was used to overexpress HtrA2. The mock and HtrA2 vector constructs were transfected into LBRM cells using an Amaxa 4D-Nucleofector X unit and the DN- program 100 according to the manufacturer’s recommendations (Lonza).

### T cell responses *in vitro*

LBRM cells transfected with mock or HtrA2 overexpression vector were cultured in 96-well plates containing 200 μl/well of complete medium, at 37 °C in a humidified 5% (v/v) CO_2_/air atmosphere. Cells were pulsed with 1 μCi of tritiated thymidine (3[H]-TdR; NEN Life Science Products Inc., Boston, MA, USA) 18 h before harvesting and were counted with an automated harvester (PHD Cell Harvester; Cambridge Technology, Inc., Cambridge, MA, USA). Results are expressed as the mean c.p.m. of triplicate samples ± SD.

### Real-time quantitative polymerase chain reaction (PCR)

Total RNA was extracted using TRIzol reagent (Molecular Research Center. Cincinnati, OH, USA). The concentration of RNA in each sample was measured using a NanoDrop ND-1000 (Thermo Fisher Scientific, MA, USA). Total RNA (2μg) was reverse transcribed into cDNA using the Transcriptor First Strand cDNA Synthesis Kit (Roche Applied Science). Gene expression were estimated using a LightCycler 2.0 instrument (Roche Diagnostic, Mannheim, Germany) and ver. 4.0 software. All reactions were performed with LightCycler FastStart DNA Master SYBR Green I (Takara, Shiga, Japan) following the manufacturer’s instructions. Relative mRNA levels were normalized to that of β-actin. The primer sequences used to amplify the mouse genes are listed in Supplementary Table 1.

### Protein purification

The pGST-STAT3 and pGST-HtrA2 plasmids were grown overnight in 20 ml LB containing 100 μg/ml ampicillin (LBAmp). The cultures were transferred to a fresh LBAmp medium and grown for an additional 90 min at 37 °C, followed by inducing the GST-fusion proteins with 0.5 mM isopropyl-β-D-1 thiogalactopyranoside for 2 additional hours. Cells were harvested and resuspended in 1 ml EBC buffer [50 mM Tris-HCl (pH 8.0), 120 mM NaCl, and 0.5% NP40] containing 100 μg/ml lysozyme. The cell suspension was lysed by sonication for 30 sec on ice and the lysates were collected by centrifugation at 12,000 rpm for 1 min. The GST-fusion proteins were purified from the cell lysates under non-denaturing conditions by selective binding to glutathione-Sepharose beads for 20 min at room temperature. Protein quantity and purity were analyzed by comparing with bovine serum albumin of known concentrations by sodium dodecyl sulfate- polyacrylamide gel electrophoreses (SDS-PAGE), followed by Coomassie Brilliant Blue staining (0.1% Brilliant Blue R, 45% methanol, and 10% acetic acid).

### *In vitro* cleavage assay

GST-HtrA2 (0.2 μM) was incubated with GST-STAT3 (1 μM) in 100 μl of cleavage buffer [50 mM Tris-HCl (pH 7.5), 1 mM DTT] for 16 hr at 37 °C for the *in vitro* cleavage reaction. The proteins bound to the beads and the supernatant were separated by centrifugation at 12,000 rpm for 5 min and resolved by 15% SDS-PAGE and analyzed by IB assays.

### Statistical analysis

All data are expressed as mean ± standard deviation. The statistical analysis was performed using SPSS 10.0 for Windows (IBM Corp., Armonk, NY, USA). Numerical data of the groups were compared using one-way analysis of variance (ANOVA) and the nonparametric Mann–Whitney test. Differences in the mean values of various groups were analyzed by ANOVA with a post-hoc test. P-values <0.05 were statistically significant.

## Additional Information

**How to cite this article**: Lee, S. H. *et al*. HtrA2 suppresses autoimmune arthritis and regulates activation of STAT3. *Sci. Rep.* **6**, 39393; doi: 10.1038/srep39393 (2016).

**Publisher's note:** Springer Nature remains neutral with regard to jurisdictional claims in published maps and institutional affiliations.

## Supplementary Material

Supplementary Information

Supplementary Table 1

## References

[b1] Niu Q., Cai B., Huang Z. C., Shi Y. Y. & Wang L. L. Disturbed Th17/Treg balance in patients with rheumatoid arthritis. Rheumatology international 32, 2731–2736, doi: 10.1007/s00296-011-1984-x (2012). 21809006

[b2] van Hamburg J. P. . Th17 cells, but not Th1 cells, from patients with early rheumatoid arthritis are potent inducers of matrix metalloproteinases and proinflammatory cytokines upon synovial fibroblast interaction, including autocrine interleukin-17A production. Arthritis Rheum 63, 73–83, doi: 10.1002/art.30093 (2011). 20954258

[b3] Smolen J. S. . Pro-inflammatory cytokines in rheumatoid arthritis: pathogenetic and therapeutic aspects. Clinical reviews in allergy & immunology 28, 239–248, doi: 10.1385/CRIAI:28:3:239 (2005). 16129908

[b4] Ziolkowska M. . High levels of IL-17 in rheumatoid arthritis patients: IL-15 triggers *in vitro* IL-17 production via cyclosporin A-sensitive mechanism. Journal of immunology 164, 2832–2838 (2000). 10.4049/jimmunol.164.5.2832 10679127

[b5] Cho M. L. . STAT3 and NF-kappaB signal pathway is required for IL-23-mediated IL-17 production in spontaneous arthritis animal model IL-1 receptor antagonist-deficient mice. Journal of immunology 176, 5652–5661 (2006). 10.4049/jimmunol.176.9.5652 16622035

[b6] Mathur A. N. . Stat3 and Stat4 direct development of IL-17-secreting Th cells. Journal of immunology 178, 4901–4907 (2007). 10.4049/jimmunol.178.8.4901 17404271

[b7] Yang X. O. . STAT3 regulates cytokine-mediated generation of inflammatory helper T cells. The Journal of biological chemistry 282, 9358–9363, doi: 10.1074/jbc.C600321200 (2007). 17277312

[b8] O’Shea J. J. & Paul W. E. Mechanisms underlying lineage commitment and plasticity of helper CD4+ T cells. Science 327, 1098–1102, doi: 10.1126/science.1178334 (2010). 20185720 PMC2997673

[b9] Son H. J. . Metformin attenuates experimental autoimmune arthritis through reciprocal regulation of Th17/Treg balance and osteoclastogenesis. Mediators of inflammation 2014, 973986, doi: 10.1155/2014/973986 (2014). 25214721 PMC4158168

[b10] Park J. S. . STA-21, a promising STAT-3 inhibitor that reciprocally regulates Th17 and Treg cells, inhibits osteoclastogenesis in mice and humans and alleviates autoimmune inflammation in an experimental model of rheumatoid arthritis. Arthritis & rheumatology 66, 918–929, doi: 10.1002/art.38305 (2014). 24757144

[b11] Prajapati R., Plant D. & Barton A. Genetic and genomic predictors of anti-TNF response. Pharmacogenomics 12, 1571–1585, doi: 10.2217/pgs.11.114 (2011). 22044414

[b12] Hetland M. L. . Direct comparison of treatment responses, remission rates, and drug adherence in patients with rheumatoid arthritis treated with adalimumab, etanercept, or infliximab: results from eight years of surveillance of clinical practice in the nationwide Danish DANBIO registry. Arthritis and rheumatism 62, 22–32, doi: 10.1002/art.27227 (2010). 20039405

[b13] Paula F. S. & Alves J. D. Non-tumor necrosis factor-based biologic therapies for rheumatoid arthritis: present, future, and insights into pathogenesis. Biologics: targets & therapy 8, 1–12, doi: 10.2147/BTT.S35475 (2014). 24353404 PMC3861294

[b14] Vande Walle L., Lamkanfi M. & Vandenabeele P. The mitochondrial serine protease HtrA2/Omi: an overview. Cell death and differentiation 15, 453–460, doi: 10.1038/sj.cdd.4402291 (2008). 18174901

[b15] Martins L. M. . Neuroprotective role of the Reaper-related serine protease HtrA2/Omi revealed by targeted deletion in mice. Molecular and cellular biology 24, 9848–9862, doi: 10.1128/MCB.24.22.9848-9862.2004 (2004). 15509788 PMC525490

[b16] Rathke-Hartlieb S. . Progressive loss of striatal neurons causes motor dysfunction in MND2 mutant mice and is not prevented by Bcl-2. Experimental neurology 175, 87–97, doi: 10.1006/exnr.2002.7868 (2002). 12009762

[b17] Oliveira R. D. . Differential gene expression profiles may differentiate responder and nonresponder patients with rheumatoid arthritis for methotrexate (MTX) monotherapy and MTX plus tumor necrosis factor inhibitor combined therapy. The Journal of rheumatology 39, 1524–1532, doi: 10.3899/jrheum.120092 (2012). 22753658

[b18] Jones J. M. . Loss of Omi mitochondrial protease activity causes the neuromuscular disorder of mnd2 mutant mice. Nature 425, 721–727, doi: 10.1038/nature02052 (2003). 14534547

[b19] Kang S. . Loss of HtrA2/Omi activity in non-neuronal tissues of adult mice causes premature aging. Cell death and differentiation 20, 259–269, doi: 10.1038/cdd.2012.117 (2013). 22976834 PMC3554338

[b20] Jones J. M. . mnd2: a new mouse model of inherited motor neuron disease. Genomics 16, 669–677, doi: 10.1006/geno.1993.1246 (1993). 8325640

[b21] Kulkarni A. B. . Transforming growth factor beta 1 null mutation in mice causes excessive inflammatory response and early death. Proceedings of the National Academy of Sciences of the United States of America 90, 770–774 (1993). 8421714 10.1073/pnas.90.2.770 PMC45747

[b22] Tao Z. . JAK2/STAT3 pathway mediating inflammatory responses in heatstroke-induced rats. International journal of clinical and experimental pathology 8, 6732–6739 (2015). 26261556 PMC4525890

[b23] Oh H. M. . STAT3 protein promotes T-cell survival and inhibits interleukin-2 production through up-regulation of Class O Forkhead transcription factors. The Journal of biological chemistry 286, 30888–30897, doi: 10.1074/jbc.M111.253500 (2011). 21730069 PMC3162449

[b24] Sato K. . Th17 functions as an osteoclastogenic helper T cell subset that links T cell activation and bone destruction. The Journal of experimental medicine 203, 2673–2682, doi: 10.1084/jem.20061775 (2006). 17088434 PMC2118166

[b25] Thomson T. M. . Blood-based identification of non-responders to anti-TNF therapy in rheumatoid arthritis. BMC medical genomics 8, 26, doi: 10.1186/s12920-015-0100-6 (2015). 26036272 PMC4455917

[b26] Nakashima Y., Plump A. S., Raines E. W., Breslow J. L. & Ross R. ApoE-deficient mice develop lesions of all phases of atherosclerosis throughout the arterial tree. Arteriosclerosis and thrombosis: a journal of vascular biology / American Heart Association 14, 133–140 (1994). 10.1161/01.atv.14.1.133 8274468

[b27] Reddick R. L., Zhang S. H. & Maeda N. Atherosclerosis in mice lacking apo E. Evaluation of lesional development and progression. Arteriosclerosis and thrombosis: a journal of vascular biology / American Heart Association 14, 141–147 (1994). 10.1161/01.atv.14.1.141 8274470

[b28] Wei L., Laurence A., Elias K. M. & O’Shea J. J. IL-21 is produced by Th17 cells and drives IL-17 production in a STAT3-dependent manner. The Journal of biological chemistry 282, 34605–34610, doi: 10.1074/jbc.M705100200 (2007). 17884812 PMC2323680

[b29] Koenders M. I., Joosten L. A. & van den Berg W. B. Potential new targets in arthritis therapy: interleukin (IL)-17 and its relation to tumour necrosis factor and IL-1 in experimental arthritis. Annals of the rheumatic diseases 65 Suppl 3, iii29–33, doi: 10.1136/ard.2006.058529 (2006). 17038468 PMC1798387

[b30] Wang L. . IL-17 can promote tumor growth through an IL-6-Stat3 signaling pathway. The Journal of experimental medicine 206, 1457–1464, doi: 10.1084/jem.20090207 (2009). 19564351 PMC2715087

[b31] Hwang S. Y. . IL-17 induces production of IL-6 and IL-8 in rheumatoid arthritis synovial fibroblasts via NF-kappaB- and PI3-kinase/Akt-dependent pathways. Arthritis research & therapy 6, R120–128, doi: 10.1186/ar1038 (2004). 15059275 PMC400429

[b32] Cho M. L. . IL-17 induces the production of IL-16 in rheumatoid arthritis. Experimental & molecular medicine 40, 237–245, doi: 10.3858/emm.2008.40.2.237 (2008). 18446062 PMC2679298

[b33] Kim K. W., Kim H. R., Kim B. M., Cho M. L. & Lee S. H. Th17 Cytokines Regulate Osteoclastogenesis in Rheumatoid Arthritis. The American journal of pathology 185, 3011–3024, doi: 10.1016/j.ajpath.2015.07.017 (2015). 26362732

[b34] Newcomb D. C. . IL-13 regulates Th17 secretion of IL-17A in an IL-10-dependent manner. Journal of immunology 188, 1027–1035, doi: 10.4049/jimmunol.1102216 (2012). PMC3262924 22210911

[b35] de Vries J. E. The role of IL-13 and its receptor in allergy and inflammatory responses. The Journal of allergy and clinical immunology 102, 165–169 (1998). 9723655 10.1016/s0091-6749(98)70080-6

[b36] Laidlaw B. J., Craft J. E. & Kaech S. M. The multifaceted role of CD4(+) T cells in CD8(+) T cell memory. Nature reviews. Immunology 16, 102–111, doi: 10.1038/nri.2015.10 (2016). PMC4860014 26781939

[b37] Gerberick G. F., Cruse L. W., Miller C. M., Sikorski E. E. & Ridder G. M. Selective modulation of T cell memory markers CD62L and CD44 on murine draining lymph node cells following allergen and irritant treatment. Toxicology and applied pharmacology 146, 1–10, doi: 10.1006/taap.1997.8218 (1997). 9299591

[b38] Sprecher D. L. . Cardiovascular features of homozygous familial hypercholesterolemia: analysis of 16 patients. The American journal of cardiology 54, 20–30 (1984). 6331147 10.1016/0002-9149(84)90298-4

[b39] Cush J. J. . Elevated interleukin-10 levels in patients with rheumatoid arthritis. Arthritis and rheumatism 38, 96–104 (1995). 7818579 10.1002/art.1780380115

[b40] Bhattacharya P. . GM-CSF: An immune modulatory cytokine that can suppress autoimmunity. Cytokine 75, 261–271, doi: 10.1016/j.cyto.2015.05.030 (2015). 26113402 PMC4553090

[b41] Bhattacharya P. . Dual Role of GM-CSF as a Pro-Inflammatory and a Regulatory Cytokine: Implications for Immune Therapy. Journal of interferon & cytokine research: the official journal of the International Society for Interferon and Cytokine Research 35, 585–599, doi: 10.1089/jir.2014.0149 (2015). PMC4529096 25803788

[b42] Rowin J. . Granulocyte macrophage colony-stimulating factor treatment of a patient in myasthenic crisis: effects on regulatory T cells. Muscle & nerve 46, 449–453, doi: 10.1002/mus.23488 (2012). 22907239 PMC3428740

[b43] Campbell I. K. . Protection from collagen-induced arthritis in granulocyte-macrophage colony-stimulating factor-deficient mice. Journal of immunology 161, 3639–3644 (1998). 9759887

[b44] Farooq S. M., Kumar A. & Ashour H. M. Eye-mediated immune tolerance to Type II collagen in arthritis-prone strains of mice. Journal of cellular and molecular medicine 18, 2512–2518, doi: 10.1111/jcmm.12376 (2014). 25211510 PMC4302655

[b45] Farooq S. M. & Ashour H. M. Type II collagen induces peripheral tolerance in BALB/c mice via the generation of CD8+ T regulatory cells. PloS one 7, e48635, doi: 10.1371/journal.pone.0048635 (2012). 23133648 PMC3487721

[b46] Hyun-Ah S. . A Simple and Accurate Genotype Analysis of the motor neuron degeneration 2 (mnd2) Mice: an Easy-to-Follow Guideline and Standard Protocol Applicable to Mutant Mouse Models. IBC 4, 8, doi: 10.4051/ibc.2012.4.3.0008 (2012).

[b47] Williams R. O., Feldmann M. & Maini R. N. Anti-tumor necrosis factor ameliorates joint disease in murine collagen-induced arthritis. Proceedings of the National Academy of Sciences of the United States of America 89, 9784–9788 (1992). 1409699 10.1073/pnas.89.20.9784 PMC50217

