# Supplementary material for: HtrA2 suppresses autoimmune arthritis and regulates activation of STAT3
Source: Sci Rep. 2016 Dec 23;6:39393. doi: 10.1038/srep39393 (PMC5180098; doi:10.1038/srep39393)
Supplement: Supplementary Information [file srep39393-s1.pdf]

HtrA2 suppresses autoimmune arthritis and regulates activation of STAT3

Running title: HtrA2 inhibits STAT3 activation in CIA

Seung Hoon Lee[1*], Young-Mee Moon[1, 2*], Hyeon-Beom Seo[1*], Se-Young Kim[1], Eun-Kyung Kim[1], Junyeong Yi [3, 4], Min-Kyung Nam[3, 4], Jun-Ki Min[1], Sung-Hwan Park[1], Hyangshuk Rhim[3, 4] and Mi-La Cho[1, 2]

[1]The Rheumatism Research Center, Catholic Research Institute of Medical Science, The Catholic University of Korea, Seoul, South Korea

[2]Laboratory of Immune Network, Conversant Research Consortium in Immunologic disease, College of Medicine, The Catholic University of Korea

[3]Department of Biomedicine and Health Sciences, College of Medicine, The Catholic University of Korea, Seoul 137-701, Republic of Korea

[4]Department of Medical Life Sciences, College of Medicine, The Catholic University of Korea, Seoul 137-701, Republic of Korea

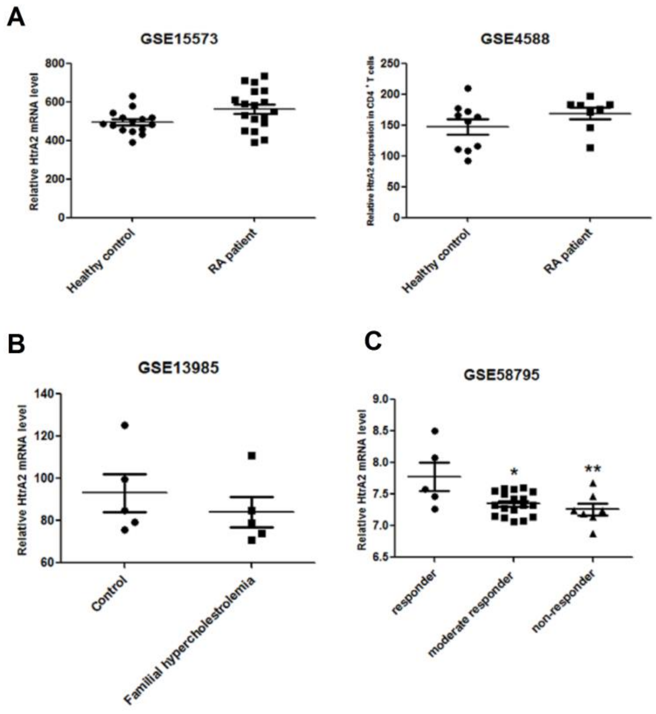

Supplementary Figure 1. HtrA2 is associated with non-response of RA. (A) Gene expression of HtrA2 in peripheral blood mononuclear cells of healthy individual and RA patients. (B) HtrA2 gene level in $CD4^+$ T cells sorted from peripheral blood mononuclear cells of healthy individuals and RA patients. (C) Gene expression of HtrA2 in peripheral blood mononuclear cells of healthy control and patients with familial hypercholesterolemia. (D) HtrA2 gene level in peripheral blood mononuclear cells of responder, moderate responder and non-responder of RA patients. Data are presented as the mean ± SD (*P < 0.05, **P < 0.03).
