# Supplementary material for: HtrA2 suppresses autoimmune arthritis and regulates activation of STAT3
Source: Sci Rep. 2016 Dec 23;6:39393. doi: 10.1038/srep39393 (PMC5180098; doi:10.1038/srep39393)
Supplement: Supplementary Table 1 [file srep39393-s2.pdf]

HtrA2 suppresses autoimmune arthritis and regulates activation of STAT3

Running title: HtrA2 inhibits STAT3 activation in CIA

Seung Hoon Lee[1*], Young-Mee Moon[1, 2*], Hyeon-Beom Seo[1*], Se-Young Kim[1], Eun-Kyung Kim[1], Junyeong Yi [3, 4], Min-Kyung Nam[3, 4], Jun-Ki Min[1], Sung-Hwan Park[1], Hyangshuk Rhim[3, 4] and Mi-La Cho[1, 2]

[1]The Rheumatism Research Center, Catholic Research Institute of Medical Science, The Catholic University of Korea, Seoul, South Korea

[2]Laboratory of Immune Network, Conversant Research Consortium in Immunologic disease, College of Medicine, The Catholic University of Korea

[3]Department of Biomedicine and Health Sciences, College of Medicine, The Catholic University of Korea, Seoul 137-701, Republic of Korea

[4]Department of Medical Life Sciences, College of Medicine, The Catholic University of Korea, Seoul 137-701, Republic of Korea

Gene	Sense primer (5'-->3)	Antisense primer (3'-->5')
PTEN	AAT TCC CAG TCA GAG GCG CTA TGT	GAT TGC AAG TTC CGC CAC TGA ACA
IL-17A	CCT CAA AGC TCA GCG TGT CC	GAG CTC ACT TTT GCG CCA AG
CCL20	CAG CTG TTG CCT CTC GTA CA	CAC CCA GTT CTG CTT TGG AT
RORγt	TGT CCT GGG CTA CCC TAC TG	GTC CAG GAG TAG GCC ACA TT
BATF	GAC AAG AAG GGC GAT GCT AC	GGC CTT AAG TCC CTC TGA CC
RUNX1	TAC CTG GGA TCC ATC ACC TC	GAC GGC AGA GTA GGG AAC TG
Foxp3	GGC CCT TCT CCA GGA CAG A	GCT GAT CAT GGC TGG GTT GT
SOCS3	CCT TTG ACA AGC GGA CTC TC	GCC AGC ATA AAA ACC CTT CA
Bcl2	GCATCCCAGCCTCCGTTAT	TGAGTACCTGAACCGGCATCT
β-actin	GAA ATC GTG CGT GAC ATC AAA G	TGT AGT TTC ATG GAT GCC ACA G

Supplementary table 1. PCR primer sequence used in this study.
